# Engineering of CRISPR/Cas9‐mediated potyvirus resistance in transgene‐free *Arabidopsis* plants

**DOI:** 10.1111/mpp.12417

**Published:** 2016-06-27

**Authors:** Douglas E. Pyott, Emma Sheehan, Attila Molnar

**Affiliations:** ^1^ Institute of Molecular Plant Sciences, University of Edinburgh Edinburgh EH9 3JR UK

**Keywords:** CRISPR/Cas9, *eIF(iso)4E*, potyvirus, transgene‐free, TuMV, virus resistance

## Abstract

Members of the eukaryotic translation initiation factor (eIF) gene family, including *eIF4E* and its paralogue *eIF(iso)4E*, have previously been identified as recessive resistance alleles against various potyviruses in a range of different hosts. However, the identification and introgression of these alleles into important crop species is often limited. In this study, we utilise CRISPR/Cas9 technology to introduce sequence‐specific deleterious point mutations at the *eIF(iso)4E* locus in *Arabidopsis thaliana* to successfully engineer complete resistance to *Turnip mosaic virus* (TuMV), a major pathogen in field‐grown vegetable crops. By segregating the induced mutation from the *CRISPR/Cas9* transgene, we outline a framework for the production of heritable, homozygous mutations in the transgene‐free T_2_ generation in self‐pollinating species. Analysis of dry weights and flowering times for four independent T_3_ lines revealed no differences from wild‐type plants under standard growth conditions, suggesting that homozygous mutations in *eIF(iso)4E* do not affect plant vigour. Thus, the established CRISPR/Cas9 technology provides a new approach for the generation of *Potyvirus* resistance alleles in important crops without the use of persistent transgenes.

## Introduction

Plant viruses are ubiquitous in natural environments and can severely limit plant growth and fertility. Globally, viruses are a significant economic burden to both well‐developed and under‐developed agriculture because of absolute yield losses in the field and decreased marketability of harvested crops. The *Potyvirus* genus contains a greater number of virus species than any other plant virus genus (Gibbs and Ohshima, 2010), and certain species within this genus [notably its type member, *Potato virus Y* (PVY)] are particularly damaging to economically important crops (Karasev and Gray, 2013). Potyviruses exist as flexuous, rod‐shaped virions comprising a positive‐sense, single‐stranded RNA (+ssRNA) genome coated by a virally encoded coat protein (Jagadish *et al*., 1991). On entry into plant cells, the +ssRNA genome is uncoated and translated into a single polypeptide, which subsequently generates the range of potyviral proteins by autocatalysis (Carrington *et al*., 1989). Translation of the potyviral RNA is largely dependent on host translation factors.

In eukaryotes, translation of mRNAs is orchestrated by multi‐component translation complexes composed of eukaryotic initiation factors (eIFs), which recruit ribosomes to the 5′ untranslated region (UTR). eIF4E and eIF4G associate to form an eIF4F core complex. eIF4G acts as a scaffold protein which associates with the DEAD box RNA helicase eIF4A, and the polyA‐binding protein (PABP), which unwind and circularize the mRNA, respectively. eIF4E associates with the 5′ m^7^GpppN cap structure, which is crucial for mRNA circularization and anchoring of the translation complex to the 5′ UTR. In higher plants, gene duplication results in a second eIF4F complex, called eIF(iso)4F, which is composed of eIF(iso)4E and eIF(iso)4G (Browning, 1996). Although eIF4F and eIF(iso)4F are usually formed by cognate pairing of their respective subunits [eIF4E/eIF4G and eIF(iso)4E/eIF(iso)4G] (Bush *et al*., 2009), functional redundancy exists, such that single mutations in one complex can be compensated for by activity of the other (Duprat *et al*., 2002).

The potyviral ‘VPg’ (viral protein genome‐linked) protein is involved in the usurpation of host translation complexes to aid viral translation. This is partly achieved by VPg interacting with both the 5′ UTR of the viral genome and host eIFs [such as eIF4E or eIF(iso)4E]. In contrast with cellular mRNAs, which can utilize both eIF4E and eIF(iso)4E, different potyviral VPgs have evolved to bind specifically to one or other isoform. This exclusive binding was first demonstrated for the VPg of *Turnip mosaic virus* (TuMV) in a yeast two‐hybrid screen, which showed VPg interaction with *Arabidopsis* eIF(iso)4E, but not eIF4E (Wittmann *et al*., 1997). Later, it was shown that this specific requirement for eIF(iso)4E could result in TuMV resistance if both alleles of *eIF(iso)4E* were knocked out (Duprat *et al*., 2002; Lellis *et al*., 2002; Sato *et al*., 2005). More broadly, this exclusivity of different potyviral VPgs for their cognate eIF partners has formed the basis for natural, recessive resistance to a wide range of potyviruses in various crops. For example, pepper *pvr2* (Ruffel *et al*., 2002), lettuce *mo1* (Nicaise *et al*., 2003), pea *sbm1* (Gao *et al*., 2004), tomato *pot1* (Ruffel *et al*., 2005), barley *rym4* (Kanyuka *et al*., 2005; Stein *et al*., 2005), melon *nsv* (Nieto *et al*., 2006) bean *bc‐3* (Naderpour *et al*., 2010) and potato *eva1* (Duan *et al*., 2012) have all been mapped to either *eIF4E* or *eIF(iso)4E* homologues.

The field of site‐specific genome editing has been revolutionized recently by the discovery and characterization of a programmable, RNA‐guided DNA endonuclease from *Streptococcus pyrogenes*, called Cas9, which operates together with CRISPR (*C*lustered *R*egularly *I*nterspaced *P*alindromic *R*epeats) loci in the bacterial genome as a form of defence against invading plasmids or DNA viruses in bacteria (Jinek *et al*., 2012). In its natural context, Cas9 associates with a so‐called crRNA (CRISPR RNA), derived from the invading DNA, and a tracrRNA (trans‐acting CRISPR RNA), which associate with the Cas9 protein. Cas9 is guided to the invading DNA by the crRNA via Watson–Crick base pairing, and it destroys the invading sequences by introducing double‐stranded breaks (DSBs) at a specific site. A breakthrough in the application of CRISPR/Cas9 came from the discovery that Cas9 can be programmed to introduce DSBs in eukaryotic cells guided by an artificial sgRNA (single guide RNA), which fuses components of the crRNA and tracrRNA (Jinek *et al*., 2012). The only constraint on the design of the sgRNA is that the 20‐nucleotide region of complementarity between the sgRNA and the target DNA must be immediately upstream of an NGG sequence (where ‘N’ is any base), known as a PAM (*p*roto‐spacer *a*djacent *m*otif). Introduction of sgRNA‐programmed Cas9 into eukaryotic cells leads to Cas9‐induced DSBs in the target DNA specifically three nucleotides upstream of the PAM (Gasiunas *et al*., 2012; Jinek *et al*., 2012). As these DSBs are primarily repaired by the error‐prone non‐homologous end‐joining (NHEJ) DNA repair pathway, Cas9‐induced DSBs will often result in short insertions/deletions (indels) at the site of DNA cleavage. As such, CRISPR/Cas9 technology has opened up a facile means for the introduction of site‐specific mutations in eukaryotic genomes. Since its watershed publication, CRISPR/Cas9 technology has been adopted in various model organisms, including zebrafish (Hwang *et al*., 2013), mouse (Wang *et al*., 2013), rat (Li *et al*., 2013), *Arabidopsis thaliana* (Feng *et al*., 2013) and *Nicotiana benthamiana* (Nekrasov *et al*., 2013). Importantly, CRISPR/Cas9 has recently been used to modify genomes of several crop plants (Brooks *et al*., 2014; Cai *et al*., 2015; Ito *et al*., 2015; Jacobs *et al*., 2015; Jiang *et al*., 2013; Liang *et al*., 2014; Shan *et al*., 2013, 2014; Zhou *et al*., 2014; Zhu *et al*., 2016), although most of these studies have been proofs of concept, and there are few examples to date in which the technology has been used for crop improvement (Ito *et al*., 2015).

As loss‐of‐function mutations in components of the eIF4F translation complex have repeatedly been associated with stable resistance to several potyviruses, we aimed to generate virus‐resistant plants by novel mutation at the *eIF(iso)4E* locus in *Arabidopsis thaliana* using CRISPR/Cas9 technology. Our rationale for the induction of such mutations by CRISPR/Cas9 genome editing was to showcase the concept for the generation of virus resistance, which can be applied directly to important crops in the future. As CRISPR/Cas9 has been shown to be a viable technology for site‐specific genome editing in several plant species, we believe that this work will pave the way as a strategy for the reverse engineering of potyviral resistance in a wide variety of crops.

## Results

### Site‐specific mutation of *eIF(iso)4E* by transgenic expression of an sgRNA‐guided Cas9

**Figure 1:**
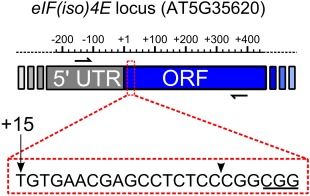
Schematic diagram of the *eIF(iso)4E* locus targeted for editing by CRISPR/Cas9. Primers flanking the target site are shown by half arrows over the 5′ untranslated region (UTR) and open reading frame (ORF) shaded grey and blue, respectively. The scale bar depicts the approximate positions, in base pairs, relative to the translation start site (+1). The enlarged area indicated by the red broken line shows the position and sequence of the sgRNA (single guide RNA), with the protospacer adjacent motif (PAM) underlined. The position of the sgRNA relative to the +1 of the ORF is indicated by an arrow. The Cas9 cleavage site is indicated by an arrowhead.

We chose to target *eIF(iso)4E* in *Arabidopsis* (At5G35620) for CRISPR/Cas9 mutagenesis, as mutations caused by ethyl methanesulfonate (EMS) (Lellis *et al*., 2002; Sato *et al*., 2005) and transposon insertion (Duprat *et al*., 2002) at this locus have been shown previously to result in complete resistance to several potyviruses, including TuMV (Duprat *et al*., 2002), *Lettuce mosaic virus* (LMV) (Duprat *et al*., 2002) and *Tobacco etch virus* (TEV) (Lellis *et al*., 2002). We decided to target the 5′ region of the open reading frame (ORF), as mutations here would increase the likelihood of creating non‐functional proteins by causing a coding frameshift or early stop codons. Specifically, we designed an sgRNA complementary to bases +15 to +35 relative to the translation start site of the gene (Fig. 1). This particular region was selected because it allowed the end of the target region of the sgRNA (immediately upstream of the PAM) to end in a guanine dinucleotide (GG), which has been reported to increase the efficacy of Cas9‐induced mutations at the correct target site (Farboud and Meyer, 2015). In addition, when we queried this sgRNA for possible off‐targeting, using the online search tools CRISPR‐Plant and CCTop (Stemmer *et al*., 2015), we found no off‐target loci. For this, we used search criteria which only qualified off‐targets with fewer than five mismatching bases to the sgRNA and no more than two mismatching bases in the seed region (12 consecutive nucleotides upstream of the PAM), as these parameters have been experimentally validated as the minimum requirements for directing Cas9 to cleave DNA targets (Hsu *et al*., 2013; Sternberg *et al*., 2015). We cloned this sgRNA into a binary vector (pDe‐CAS9) (Fauser *et al*., 2014), which allowed for the tandem expression of a plant codon‐optimized Cas9 and the sgRNA (driven by *PcUbi4‐2* and *AtU6‐26* promoters, respectively), together with the BASTA resistance gene. This construct was introduced into *Arabidopsis* (Col‐0 accession) plants by *Agrobacterium*‐mediated transformation (floral dipping).

**Figure 2:**
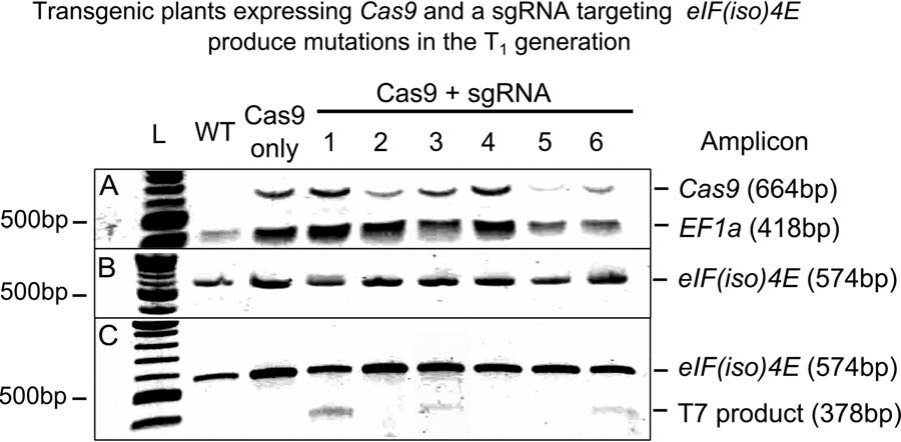
Polymerase chain reaction (PCR)/T7 endonuclease products for six independently transformed plants (T_1_ generation) containing a Cas9 transgene with an sgRNA (single guide RNA) targeting the *eIF(iso)4E* locus. Transformation with a *Cas9* transgene with no sgRNA (Cas9 only) and a non‐transformed wild‐type (WT) plant were used as controls. L denotes a 100‐bp DNA ladder. (A) A 1% agarose gel showing multiplex PCR products confirming the presence/absence of the *Cas9* transgene, using the constitutively expressed house‐keeping gene *EF1a* as a loading control. (B) A 1% agarose gel showing PCR amplicons spanning the putative mutation site at the *eIF(iso)4E* locus. (C) A 2% agarose gel showing *eIF(iso)4E* cleavage products after a self‐annealing reaction of the PCR amplicon, and subsequent digestion with T7 endonuclease. The presence of the 378‐bp cleavage product in samples 1, 3 and 6 is indicative of Cas9‐induced mutation at the *eIF(iso)4E* locus (the corresponding 196‐bp cleavage product is not visible on the gel).

Transgenic T_1_ seeds were BASTA selected, and six healthy‐looking plants were chosen to test for genome editing at the *eIF(iso)4E* locus. For this, we adopted an assay which allows edited sequences to be detected on a gel after incubation with T7 endonuclease (hereafter T7). Briefly, primers spanning the target site (Fig. 1) were used to generate an *eIF(iso)4E* amplicon, which was subsequently denatured and re‐annealed before incubation with T7. Bulges caused by mismatching bases are recognized by T7, which results in cleavage of the re‐annealed amplicons. Hence, samples with a mixture of wild‐type and mutant DNA will yield cleavage products which can be resolved on a gel, whereas homogeneous samples of either wild‐type or fully mutated DNA will be resistant to T7 activity, yielding a full, uncleaved target amplicon. A possible drawback of this assay is that samples with identical lesions in all DNA copies will be indistinguishable from the wild‐type, and hence such samples would be missed in a screen for detecting mutations. However, we reasoned that this would be a highly unlikely scenario in the T_1_ generation as Cas9 expression could only have been present in one of the parental gametes and, as such, genome editing at this stage would most likely result in chimeric plants containing a mixture of wild‐type and mutant sequences. We performed the T7 assay on *eIF(iso)4E* amplicons from six BASTA‐selected T_1_ transformants alongside a wild‐type, non‐transformed control and a T_1_ plant transformed with a Cas9 vector lacking an sgRNA sequence (Fig. 2). T7 cleavage products were absent from both negative controls, but were clearly visible in candidate plants numbered 1, 3 and 6 (Fig. 2C). As the signal for the T7 cleavage product was strongest in sample number 1, we selected this line for further analysis.

### Segregation of the induced mutation from the transgene in the T_2_ generation

**Figure 3:**
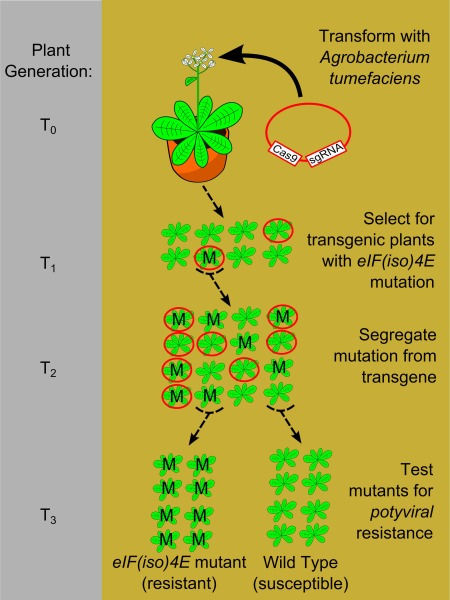
Schematic diagram of project workflow. *Arabidopsis* Col‐0 plants were transformed by floral dipping with the *Cas9/sgRNA* recombinant binary vector containing a BASTA resistance gene [pDe‐Cas9‐sgAteIF(iso)4E]. After self‐pollination, the seeds were collected and germinated in soil. Plants carrying the transgenic construct (red circle) were selected in the T_1_ generation by spraying with BASTA. The transgenic plants were then tested for *eIF(iso)4E* mutation (M) to identify plants with an active CRISPR/Cas9 nuclease using a T7 assay. One transgenic T_1_ plant with clear signs of *eIF(iso)4E* editing was used to produce the T_2_ generation. Polymerase chain reaction (PCR) was used to identify T_2_ generation plants which had lost the transgene by Mendelian segregation. The non‐transgenic T_2_ plants were then screened for *eIF(iso)4*E mutations by Sanger sequencing. (Please note: the mutations depicted as ‘M’ in the diagram are not identical, as the mutations in the T_1_ generation occurred in somatic cells, and so were not heritable, and different mutations were recovered in the T_2_ generation because of independent editing events in the germline of T_1_. For simplicity, ‘M’ was used to depict all CRISPR/Cas9‐induced mutations.) Non‐transgenic T_2_ plants, which were homozygous for either the mutated or wild‐type *eIF(iso)4e* alleles, were used to produce T_3_ populations, which were then tested for viral resistance.

**Figure 4:**
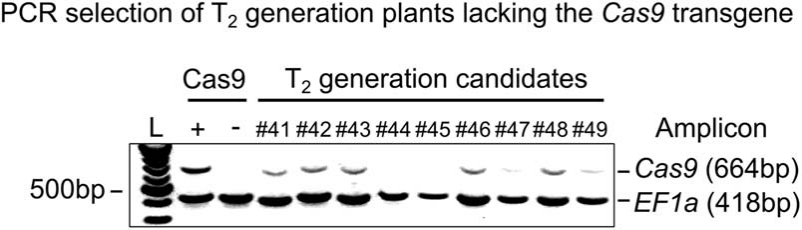
Representative 1% agarose gel for the selection of T_2_ candidates lacking the *Cas9* transgene. Multiplex polymerase chain reaction (PCR) was used to confirm the presence/absence of the *Cas9* transgene, using the constitutively expressed house‐keeping gene *EF1a* as a loading control. L denotes a 100‐bp DNA ladder. A Cas9 transformant (T_1_ generation) and a non‐transformed wild‐type plant were used as positive and negative controls for *Cas9* amplification, respectively. Samples #41–#49 are a representative selection of T_2_ progeny from T_1_ plant number 1 (as shown in Fig. 2). Candidates #44 and #45 represent two of a total of 55 candidates lacking the *Cas9* transgene, which were selected by this method.

**Figure 5:**
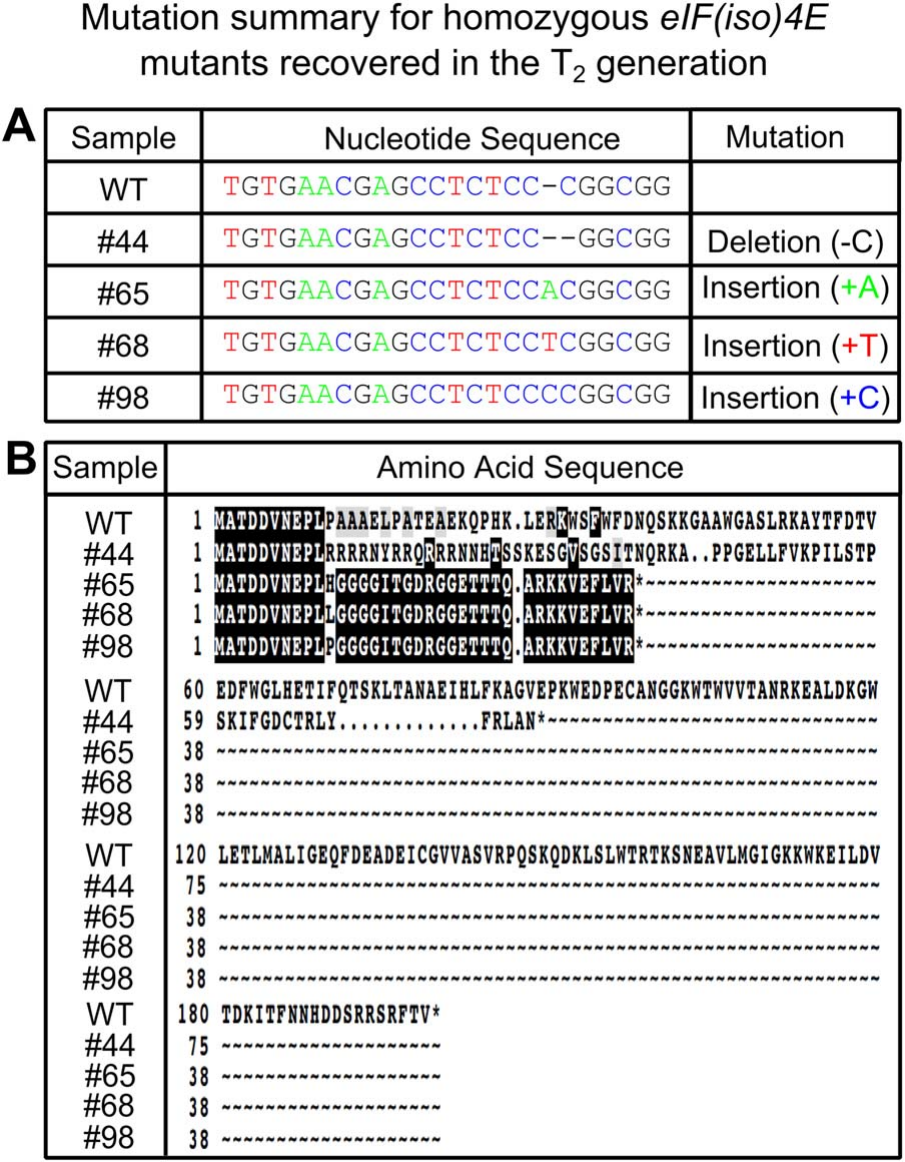
Summary of CRISPR/Cas9‐induced *eIF(iso)4E* mutations. (A) DNA sequence alignments for the four homozygous *eIF(iso)4E* mutants (#44, #65, #68, #98) identified in the T_2_ generation, together with a wild‐type (WT) control. Lines #65, #68 and #98 exhibit single‐nucleotide insertions, whereas line #44 has a single‐nucleotide deletion. (B) Predicted amino acid sequence alignments for the four homozygous mutants and the wild‐type consensus. Each of the mutant alleles codes for severely truncated and disrupted proteins.

We generated T_2_ seeds by allowing the selected T_1_ plant (line number 1) to self‐pollinate. We reasoned that it would be possible to segregate stable, uniform *eIF(iso)4E* mutations from the transgene at this generation. To establish the number of integration sites for the transgene in this line, we sprayed approximately 200 of the T_2_ seedlings from T_1_ line number 1 with BASTA. The BASTA resistant : susceptible ratio was approximately 3 : 1, indicative of a single integration site of the transgene. In parallel, with a separate batch of segregating T_2_ seedlings, we utilized a multiplex polymerase chain reaction (PCR) screen to identify T_2_ plants which had lost the *Cas9/sgRNA*‐expressing transgene. The general strategy to segregate the transgene from the induced mutation is outlined in Fig. 3. We tested 144 plants in this way and recovered 55 non‐transgenic plants (Fig. 4). Next, we tested this population of transgene‐free plants for *eIF(iso)4E* mutations. As we hoped to identify homozygous mutants, the T7 assay was inappropriate for this analysis and, instead, we opted to directly sequence *eIF(iso)4E* amplicons for each of the 55 lines by Sanger sequencing. By carefully checking the quality of the base calls at the predicted mutation site, we were able to distinguish homozygous from heterozygous mutations, as the quality score for the latter would show a sharp fall at the mutation site because of mixed signal base calling (Fig. S1, bottom panel, see Supporting Information). Of the 55 non‐transgenic plants, 39 (70.9%) harboured mutations in *eIF(iso)4E*, and four of these mutations were found to be homozygous (Figs 5A and S1). Interestingly, the majority of all mutations were single‐nucleotide indels, with the exception of one heterozygous mutant, which had a 57‐bp deletion, spanning the predicted Cas9 cleavage site (Fig. S2, see Supporting Information). This over‐representation of single‐nucleotide indels is consistent with previous reports for Cas9‐induced editing in plants (Nekrasov *et al*., 2013). Importantly, all of the indels aligned perfectly with the expected Cas9 cleavage site, 3 bp upstream of the PAM (Fig. S1). Furthermore, mutations of interest were confirmed by re‐sequencing the *eIF(iso)4E* amplicons from the opposite direction to rule out the unlikely possibility of false positives caused by sequencing errors. Each of the homozygous mutants had early stop codons in their predicted amino acid sequences (Fig. 5B), which we reasoned would create complete functional knock‐outs by severe truncation of the eIF(iso)4E protein. These four homozygous mutants (named #44, #65, #68 and #98) were self‐pollinated to produce T_3_ populations for each of the different *eIF(iso)4E* point mutations. A non‐transgenic T_2_ plant with homozygous wild‐type *eIF(iso)4E* alleles (#105) was also selected to produce a wild‐type T_3_ population.

### The induced mutation in *eIF(iso)4E* confers complete resistance to TuMV

**Figure 6:**
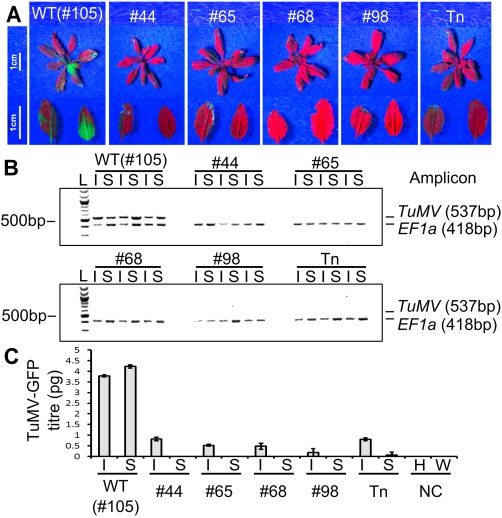
(A) Representative photographs of TuMV‐GFP (green fluorescent protein‐expressing *Turnip mosaic virus* clone)‐infected plants imaged under UV light at 7 days post‐infection. A transposon‐induced *eIF(iso)4E* mutant (Tn) was used as a resistant control. Enlarged images of inoculated (left) and systemic (right) leaves are shown below each rosette. (B) Reverse transcription‐polymerase chain reaction (RT‐PCR) to detect the presence of TuMV‐GFP in inoculated (I) and systemic (S) leaves for each genotype. Three separate plants were analysed per genotype. The first two lanes for each genotype correspond to the leaves imaged in (A). Amplicons of the TuMV coat protein region (537 bp) and the house‐keeping gene *EF1a* (418 bp) were PCR amplified separately from the same cDNA, mixed and run together on a 2% agarose gel. L denotes a 100‐bp DNA ladder. TuMV‐specific amplicons are clearly visible in each of the wild‐type (WT) samples, but completely absent from any of the *eIF(iso)4E* mutant samples. (C) Quantitative RT‐PCR to detect the mean absolute viral titre (in picograms) for the samples shown in (A) and (B). Quantitative RT‐PCRs were performed with cDNA from a healthy plant (H) and water (W) as negative controls (NC). Error bars show the standard error of the mean (SEM) of three biological replicates.

**Figure 7:**
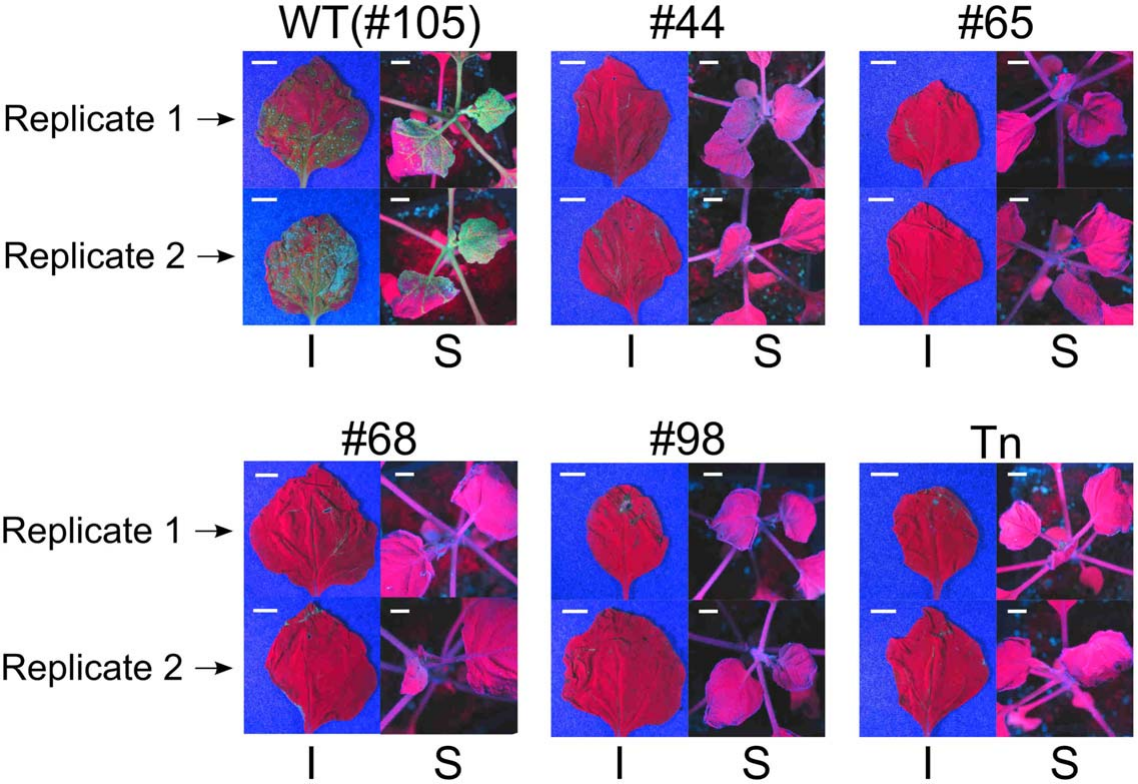
Back‐inoculations of *Nicotiana benthamiana* plants using sap from TuMV‐GFP (green fluorescent protein‐expressing *Turnip mosaic virus* clone)‐inoculated *Arabidopsis*. Sap was prepared by pooling 20 systemic leaves from TuMV‐GFP‐inoculated *Arabidopsis* plants as shown in Fig. S3. Labels above each quadrant refer to the genotype [wild‐type (WT), #44, #65, #68, #98 and transposon (Tn)] of the inoculated *Arabidopsis* used to make sap. Each quadrant shows an inoculated leaf (I) and systemic tissue (S) for two replicate plants imaged under UV light. A 1‐cm scale bar is shown for each image.

The four separate T_3_ populations (#44, #65, #68 and #98) harbouring each of the induced *eIF(iso)4E* mutations were grown alongside the T_3_ wild‐type control (#105) and a previously published transposon insertion *eIF(iso)4E* mutant, which is known to be resistant to TuMV infection (Duprat *et al*., 2002). After 4 weeks of growth, 40 plants from each of the six different genotypes (#44, #65, #68, #98, #105 and the transposon insertion mutant) were rub inoculated with a green fluorescent protein (GFP)‐expressing TuMV clone (hereafter TuMV‐GFP) (Garcia‐Ruiz *et al*., 2010). At 7 and 14 days post‐inoculation (dpi), TuMV infection was assessed by monitoring the expression of GFP in inoculated and systemic leaves (Fig. 6A). GFP expression (indicative of TuMV‐GFP infection) was clearly visible at 14 dpi in 37 of 40 (92.5%) wild‐type plants (#105), but not in any of the CRISPR/Cas9‐induced mutants (#44, #65, #68 and #98) (Fig. S3, see Supporting Information) nor in the previously reported transposon insertion mutant. To ascertain that the lack of GFP signal in the *eIF(iso)4E* mutants was caused by complete TuMV‐GFP resistance, and not just lower viral titres, inoculated and systemic leaves of each genotype were analysed by reverse transcription‐polymerase chain reaction (RT‐PCR), amplifying the coat protein‐coding region of TuMV‐GFP. TuMV‐specific amplicons were clearly detected in both inoculated and systemic leaves from three wild‐type plants, but no TuMV amplicons were detected in any samples from any of the *eIF(iso)4E* mutants (Fig. 6B). To gain a more quantitative insight into the viral titres in the inoculated plants, quantitative RT‐PCR was performed for the inoculated and systemic leaves at 7 dpi (Fig. 6C). A serial dilution of purified TuMV‐GFP RNA was included in quantitative RT‐PCR to construct a standard curve (Fig. S4, see Supporting Information) from which we could interpolate the titre (in picograms) of TuMV‐GFP in the 7‐dpi samples. In agreement with the RT‐PCR results (Fig. 6B), we were able to detect TuMV‐GFP in all of the wild‐type samples, but not in systemic leaves of the *eIF(iso)4E* mutants. Interestingly, we measured a very low titre of TuMV‐GFP in the inoculated leaves of the *eIF(iso)4E* mutants. We suspect that this TuMV‐GFP signal may be caused by residual infectious sap from the rub inoculation, although it is also possible that a low level of TuMV replication occurs in the inoculated leaves of *eIF(iso)4E* mutants. To stringently test the TuMV resistance of the *eIF(iso)4E* mutants, 20 systemic leaves of each genotype were collected at 28 dpi and pooled to make sap for back‐inoculation of *N. benthamiana* plants, which are highly susceptible to TuMV and hence would reveal the presence of very low TuMV‐GFP titres present in the sap. Five days after the back‐inoculations, TuMV‐GFP infection was clearly visible in both local and systemic leaves inoculated with sap taken from wild‐type *Arabidopsis* plants. In contrast, TuMV‐GFP was not detected at all in any of the back‐inoculations using sap prepared from the *eIF(iso)4E* mutants (Fig. 7). This indicates that the ablation of the eIF(iso)4E protein by the induced single‐nucleotide mutations in lines #44, #65, #68 and #98 renders the plants completely resistant to TuMV‐GFP in a similar manner to the previously published transposon mutant (Duprat *et al*., 2002).

### The *eIF(iso)4E* mutants show no growth defects compared with wild‐type plants when grown under standard growth conditions

**Figure 8:**
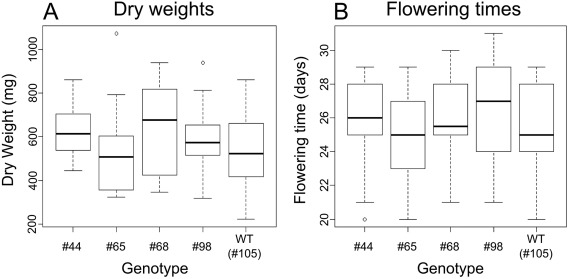
Box plots of dry weights (A) and flowering times (B) for the CRISPR/Cas9‐edited *eIF(iso)4E* mutants (lines #44, #65, #68 and #98) alongside a wild‐type (WT) plant (#105).

We noted that the *eIF(iso)4E* mutants were indistinguishable from the wild‐type plants in terms of growth. To more accurately assess the growth vigour of the *eIF(iso)4E* mutants in comparison with wild‐type plants, we measured the dry mass and flowering times of non‐infected populations of each of the four homozygous *eIF(iso)4E* mutant lines (#44, #65, #68, #98) and a wild‐type line (#105) in the T_3_ generation. For both the dry weight and flowering time experiments, seeds of each genotype were grown in a randomized block design. Ten plants from each genotype were grown together in a tray, with six replicate trays (resulting in 60 plants per genotype, spread across six different trays). The relative position of the different genotypes within any tray was randomized. These measures were taken to avoid systematic biases of the growth environment affecting one genotype more than another, and hence confounding the analysis. After 4 weeks of growth, just prior to the onset of flowering, 30 plants from each set were randomly sampled to estimate the dry weights of each genotype. The remaining plants were scored for flowering emergence by counting the number of days from germination to the first appearance of a floral bolt. From these experiments, we can conclude that there are no statistically significant differences between the growth of *eIF(iso)4E* mutants and wild‐type plants, with respect to total dry mass (*F*_4,70_ = 1.372, *P* = 0.252) or flowering time (*F*_4,119_ = 1.597, *P* = 0.180) (Fig. 8, Tables S2 and S3, see Supporting Information). From this, we surmise that this strategy of site‐specific disruption of *eIF(iso)4E* will be useful for the generation of virus‐resistant crops without concomitant constraints on plant growth.

## Discussion

In the context of a rapidly growing global population, food security is one of the major challenges facing our generation. It is estimated that food production will need to at least double in the period between 2005 and 2050 (Tilman *et al*., 2011) to meet demand. As viral infections in crops are estimated to result in approximately 10%–15% global yield reductions each year (Regenmortel and Mahy, 2009), mitigation of these losses by improved viral resistance is a worthwhile strategy to meet global yield targets. Arguably, the utilization of genetic resistance in crops is the most sustainable approach for the control of virus infections; other methods, such as pesticides to control insect vectors or manual inspection and removal of infected plants, are costly, laborious and often ineffective. There are several approaches for the introduction of resistance genes (or *R* genes) into crops. First, classical breeding can be used to identify and introgress *R* genes from related species to the crop of interest. Often wild relatives of the domesticated crop are used for these breeding programmes and successive back‐crosses are required to segregate out additional non‐beneficial (or even deleterious) alleles from the hybrid. Hence, breeding programmes can be very costly and laborious, and take many years to achieve the desired elite cultivar. Breeding programmes can also be limited by constraints such as hybrid incompatibility or outbreeding depression.

Biotechnological approaches can also be adopted to generate *R* genes in crops. A popular platform for the introduction and selection of beneficial mutations in crops is TILLING (*T*argeting *I*nduced *L*ocal *L*esions *IN G*enomes). This relies on non‐site‐specific mutation of the genome of interest, usually by EMS treatment (McCallum *et al*., 2000). The induced mutations can be subsequently identified and screened for positive attributes, such as viral resistance (Piron *et al*., 2010). This strategy overcomes some of the pitfalls of classical breeding, as it generates novel genetic diversity within the species of interest. However, this approach is also costly, time‐consuming and slow to produce elite cultivars. Furthermore, off‐target mutations caused by the random nature of the mutagenesis can result in unintended deleterious effects in the new cultivars. A second biotechnological approach to generate viral resistance is the stable integration of transgenes into crops. This transgenic approach has been utilized in a variety of ways, including: overexpression of dominant *R* genes identified in other species (Oldroyd and Staskawicz, 1998); overexpression of non‐coding sequences to direct RNA silencing of specific viruses (Asad *et al*., 2003; Bonfim *et al*., 2007; Pooggin *et al*., 2003; Yang *et al*., 2004); overexpression of null alleles to sequester functional host susceptibility factors in a dominant negative manner (Cavatorta *et al*., 2011); and stable expression of a *Cas9/sgRNA* cassette to target DNA viruses directly for CRISPR/Cas9‐mediated cleavage (Ali *et al*., 2015). Although these methods can be effective for the introduction of viral resistance into crops, transformation of plants with transgenes can be costly and laborious, and limited to only certain species or varieties with well‐established transformation protocols. Moreover, many governments are opposed to the use of transgenic products, which seriously undermines the application of these technologies for the improvement of crop yields in the near future. More recently, site‐specific genome editing technologies have offered new ways to improve crops. One attractive feature of these technologies is that, once the desired genome alterations have been made, the transgenes can be crossed out from the improved variety, thus circumventing public and political concerns around the use of persistent transgenes in crops. Early genome editing technologies include TALENs (*T*ranscription *A*ctivator‐*L*ike *E*ffector *N*ucleases) and ZFNs (*Z*inc *F*inger *N*ucleases) (Gaj *et al*., 2013). Both TALENS and ZFNs combine DNA nucleases (such as FokI) with a DNA‐binding protein to induce DNA DSBs at specific sites. A major limitation of these technologies is that tailoring the DNA‐binding proteins to target a sequence of interest can be costly and time‐consuming. Furthermore, it is only possible to engineer DNA‐binding proteins for certain DNA target sequences. The advent of CRISPR/Cas9 technology has revolutionized the field of genome editing. Crucially, the fact that the Cas9 nuclease is guided by RNA rather than protein overcomes the major limitations of TALEN and ZFN technologies. RNA‐based guiding is cheaper and easier to engineer and the range of possible target sequences is greatly expanded, requiring only the commonly occurring NGG PAM sequence.

In this study, we have showcased the utility of CRISPR/Cas9 technology for the generation of novel genetic resistance to TuMV in *Arabidopsis* by the deletion of a host factor [eIF(iso)4E] which is strictly required for viral survival. We hope that this work will pave the way for a similar strategy to be adopted in important crop species and thus provide an alternative, novel strategy for the introduction of *R* genes. Credence for this approach is given by the fact that many natural sources of *Potyvirus* resistance rely on the same principle: loss‐of‐function mutations in host eIFs (Duan *et al*., 2012; Gao *et al*., 2004; Kanyuka *et al*., 2005; Naderpour *et al*., 2010; Nicaise *et al*., 2003; Nieto *et al*., 2006; Ruffel *et al*., 2002, 2005; Stein *et al*., 2005). Therefore, we believe that it would be hard to justify objections to the commercial application of such a strategy, as the final genome‐edited product is essentially no different to varieties carrying mutant alleles arising from ‘natural’ methods of mutagenesis. It is noteworthy that the engineered viral resistance reported here is the result of a single‐nucleotide point mutation arising from the plant's own natural DNA damage repair mechanism, namely NHEJ. Moreover, although our approach was to use transgenic delivery of the *CRISPR/Cas9* cassette, we have shown that it is feasible to segregate out the transgene from the induced mutation at the target *eIF(iso)4E* locus at an early stage to produce stable, heritable point mutations without a persistent transgene. The fact that CRISPR/Cas9 induces sequence‐specific mutations, and that there were no detected off‐targets in this study, means that we can be confident that the genetic differences between the wild‐type and mutant plants are solely at the *eIF(iso)4E* locus. This results in a better system for the investigation of the effects of the mutation on plant growth, as previous studies using EMS (Lellis *et al*., 2002; Sato *et al*., 2005) or transposon insertion (Duprat *et al*., 2002) mutagenesis are likely to be confounded by multiple off‐target genomic mutations. Our analysis did not reveal any significant differences in the growth and development of the *eIF(iso)4E* mutants compared with wild‐type plants. It is still possible that, under certain growth environments, particularly stress conditions, the *eIF(iso)4E* mutants may grow differently from wild‐type plants, although this is entirely speculative and requires further investigation. The durability of this engineered resistance also remains to be tested. It is assumed that recessive resistance arising from the loss of a host factor required by the virus will be more durable than dominant *R* genes, because of the lower selective pressures on the virus to evolve counter defence strategies (Ronde *et al*., 2014). However, resistance breaking has been reported previously for recessive *eIF(iso)4E* resistance to TuMV, possibly caused by VPg polymorphisms acting via an eIF(iso)4E‐independent pathway (Gallois *et al*., 2010). It remains to be seen whether this resistance breaking will also occur in the CRISPR/Cas9‐induced *eIF(iso)4E* mutants in this study. We hypothesize that our approach will result in a more durable resistance because of the complete absence of eIF(iso)4E protein. We hope that the coming years will provide more detailed analysis to this end, and will eventually lead to the introduction of this technique for a variety of marketable crops. Indeed, whilst preparing the manuscript, a similar approach was adopted to generate viral resistance in cucumber by CRISPR/Cas9‐induced mutation of *eIF4E* (Chandrasekaran *et al*., in press). Hence, the utilization of CRISPR/Cas9 technology may provide an efficient and publically acceptable method for crop improvement in the future.

## Experimental Procedures

### Plant growth conditions

For the growth and development experiments, plants were grown in 6 cm × 6 cm × 8 cm (length × width × depth) pots on Levington F2+S professional growth compost. Plants were grown in growth rooms at 21 ºC with 16‐h : 8‐h light : dark cycles under cool, white fluorescent bulbs at a light intensity of approximately 100 µmol/m^2^/s. T_1_ seeds were grown under the same growth conditions in 50 cm × 25 cm × 6 cm (length × width × depth) trays filled with Levington F2+S professional growth compost. For all other experiments, plants were grown in 4 cm × 4 cm × 5 cm (length × width × depth) pots on Levington F2+S professional growth compost. Plants were grown in SANYO/Panasonic, Kadoma, Osaka Prefecture, Japan growth chambers set to 21 ºC with 16‐h : 8‐h light : dark cycles with side illumination from cool, white fluorescent bulbs at a light intensity of approximately 200 µmol/m^2^/s. All seeds were stratified for 48 h in darkness at 4 ºC prior to planting.

### Guide RNA design and cloning

sgRNA was designed by CRISPR Design (http://crispr.mit.edu/) and DNA2.0 CRISPR gRNA design (https://www.dna20.com/eCommerce/cas9/input) tools. The corresponding oligos, Iso_Fw and Iso_Rv (Table S1, see Supporting Information), were annealed and cloned into *Bbs*I‐digested entry vector pEn‐Chimera (Fauser *et al*., 2014). After sequence verification, the sgRNA expression cassette was recombined into the destination vector pDe‐Cas9 by Gateway cloning (LR reaction) according to the manufacturer's instructions (Life Technologies, Carlsbad, California, USA), resulting in pDe‐Cas9‐sgAteIF(iso)4E.

### Plant transformation

*Agrobacterium tumefaciens* (strain AGL1) cells were transformed with the pDE‐CAS9 and pDE‐CAS9‐sgAteIF(iso)4E plasmids by electroporation, and transformed cultures were grown overnight in Luria‐Bertani (LB) liquid medium at 28 ºC with shaking at 230 rpm to a final optical density at 600 nm (OD_600_) of 1.0. Cells were pelleted by centrifugation at 1000 g for 20 min. Pelleted cells were washed and then resuspended in liquid LB, and OD_600_ was adjusted to 0.8. Silwet was added to the culture at a final concentration of 0.1% (v/v) and acetosyringone was added to a final concentration of 150 µm. Six‐week‐old flowering *Arabidopsis* (Col‐0 accession) plants were dipped in the *Agrobacterium* suspension for approximately 10 s. This process was repeated 7 days later.

### BASTA selection

T_1_ seeds were collected and scattered evenly on compost‐filled 70 cm × 30 cm × 6 cm (length × width × depth) trays. Seven days after germination, seedlings were sprayed with a 120 mg/L solution of BASTA by applying a fine mist of the herbicide across the entire area of the tray, using a hand‐held spray bottle. The BASTA spray treatment was repeated at 14 and 21 days after germination. Plants were grown in a temperature‐controlled glasshouse at 21 ºC with a 16‐h : 8‐h light : dark cycle and a light intensity of approximately 300 µmol/m^2^/s provided by halogen light bulbs and daylight. BASTA‐resistant plants were transplanted into 6 cm × 6 cm × 8 cm (length × width × depth) pots to produce T_2_ seed.

### PCR conditions

For the preparation of *eIF(iso)4E* PCR amplicons for the T7 assay and Sanger sequencing, Q5® High Fidelity DNA polymerase (NEB, Ipswich, Massachusetts, USA #M0491S) was used with the following reaction conditions: 95 ºC for 120 s, (95 ºC for 15 s, 57 ºC for 15 s, 72 ºC for 30 s) × 35, 72 ºC for 300 s. All other PCRs were performed using Taq DNA polymerase (NEB #M0267L) with the following reaction conditions: 95 ºC for 120 s, (95 ºC for 20 s, *T*_a_ ºC for 30 s, 72 ºC for 30 s) × 35, 72 ºC for 300 s. ‘*T*_a_’ is the primer‐pair‐specific annealing temperature listed in Table S1.

### T7 endonuclease assay

For the denaturing/annealing reaction, the total Q5 PCR product (10 µL) was mixed with 1.5 µL of 10 × NEB buffer 2 (NEB #B7002S) and 1.5 µL of water. This mixture was incubated at 95 ºC for 10 min, ramped from 95 to 85 ºC at a rate of −2 ºC/s, and then from 85 to 25 ºC at a rate of −0.3 ºC/s. T7 enodnuclease I (NEB #M0302S) was diluted to a concentration of 2 U/µL (in NEB buffer 2 #B7002S), and 2 µL of this were added to the denaturing/annealing reaction product and incubated at 37 ºC for 1 h. The total T7 digestion product (15 µL) was loaded onto a 2% agarose gel and separated at 100 V.

### Sanger sequencing

*eIF(iso)4E* amplicons were generated as described above. The PCR products were purified using a MinElute PCR Purification kit (Qiagen, Hilden, Germany). The cleaned products were sequenced directly with either eIF(iso)4E_Fw or eIF(iso)4E_Rv primers (Table S1) using BigDye (Applied Biosystems, Foster city, California, USA) according to the manufacturer's protocols.

### Viral inoculations

Infectious TuMV‐GFP sap was prepared by syringe infiltration of 4‐week‐old *N. benthamiana* plants with *A. tumefaciens* (strain AGL1) cells containing the pCB‐TuMV‐GFP plasmid (Garcia‐Ruiz *et al*., 2010). *Agrobacterium* cells were grown as described above and resuspended in infiltration medium containing 10 mm 2‐(*N*‐morpholino)ethanesulfonic acid (MES) (pH 5.6), 10 mm MgCl_2_ and 150 µm acetosyringone. The cells were incubated at room temperature without shaking for 1 h before syringe infiltration into the abaxial surface of three fully expanded leaves. Fourteen days after infiltration, systemic leaves showing viral symptoms were harvested and homogenized with a sterile mortar and pestle. The homogenate was diluted 1 : 5 (w/v) in 1 mm sodium phosphate buffer (pH 7) and frozen in 0.5‐mL aliquots at −80 ºC. *Arabidopsis/N. benthamiana* leaves were rub inoculated on the adaxial surface with 10 µL of 1 : 5‐diluted viral sap aliquots using aluminium oxide powder as an abrasive. The third and fourth oldest rosette leaves of 4‐week‐old *Arabidopsis* plants were used for rub inoculations. Second and third rounds of inoculations were performed 3 and 7 days after the initial inoculation using the fourth/fifth and sixth/seventh oldest leaves, respectively.

### Viral GFP imaging and RT‐PCR/quantitative RT‐PCR

Viral expression of GFP was monitored using hand‐held UV lamps (UVP B‐100AP Lamp, 100 W, 365 nm) and imaged using a Canon, Tokyo, Japan powershot G16 digital camera.

Total nucleic acids were purified from plant tissue by phenol–chloroform extraction as described previously (White and Kaper, 1989). Three micrograms of total nucleic acids were DNase treated using a Turbo DNAse kit (Ambion, Waltham, Massachusetts, USA) according to the manufacturer's protocol. One microgram of DNase‐treated RNA was employed for cDNA synthesis using SuperscriptII reverse transcriptase (Invitrogen, Carlsbad, California, USA) according to the manufacturer's protocol. One microlitre of cDNA was used for the PCRs with TuMV‐ and Ef1a‐specific primers as described above.

For quantitative RT‐PCR, 2 µL of cDNA was used in a 10‐µL reaction with LightCycler, (Roche) Basel, Switzerland® 480 SYBR Green I Master mix, according to the manufacturer's protocol. TuMV‐specific primers (Table S1) were used at a final concentration of 1 µm. Quantitative RT‐PCR was performed in a LightCycler® 480 machine with the following amplification reaction conditions: 95 ºC for 300 s, (95 ºC for 10 s, 60 ºC for 10 s, 72 ºC for 10 s) × 50.

### Dry weight measurements

Four‐week‐old *Arabidopsis* plants were harvested for dry weight measurements. Whole plants were prepared by carefully removing the plants and soil from the pots and soaking the plant roots in water to wash the soil from the root system. Two plants per genotype were pooled and placed in pre‐weighed waxed paper cups, giving 15 replicates of two pooled plants per genotype. The plants were dried in a Binder, Tuttlingen, Germany drying and heating chamber (Model E 28) set to 100 ºC for 48 h. The difference in weight between the empty paper cups and the paper cups with dried plants was used to estimate the dry mass of the two pooled plants.

### Statistical analysis

R software was used to perform the one‐way analyses of variance (ANOVAs).

## Supporting information

Additional Supporting Information may be found in the online version of this article at the publisher's website:

**Fig. S1** Sequencing traces for the *eIF(iso)4E* amplicon. WT, wild‐type.

**Fig. S2** Sequence of the *eIF(iso)4E* mutant with a large deletion (#21) aligned to the wild‐type (WT) *eIF(iso)4E* sequence.

**Fig. S3** Images of TuMV‐GFP (green fluorescent protein‐expressing *Turnip mosaic virus* clone)‐infected plants under UV light at 14 days post‐infection. WT, wild‐type.

**Fig. S4** (A) Cp values for quantitative reverse transcription‐polymerase chain reaction (qRT‐PCR) quantification of TuMV‐GFP (green fluorescent protein‐expressing *Turnip mosaic virus* clone) in inoculated samples, as described in Fig. 6. (B) Standard curve used to interpolate the absolute concentration of TuMV‐GFP from the measured Cp values, shown in Fig. 6. NC, negative control; WT, wild‐type.

**Table S1** List of oligonucleotides used in this study, together with the specific polymerase chain reaction (PCR) annealing temperatures used and the expected amplicon sizes.

**Table S2** Dry weight for wild‐type plants (WT, #105) and homozygous *eIF(iso)4E* mutant plants (#44, #65, #68, #98). Each dry weight value is the weight of two pooled plants.

**Table S3** Flowering times for wild‐type plants (WT, #105) and homozygous *eIF(iso)4E* mutant plants (#44, #65, #68, #98).
