# Supplementary figures and images for: Engineering of CRISPR/Cas9‐mediated potyvirus resistance in transgene‐free Arabidopsis plants
Source: Mol Plant Pathol. 2016 Jun 27;17(8):1276–88. doi: 10.1111/mpp.12417 (PMC5026172; doi:10.1111/mpp.12417)

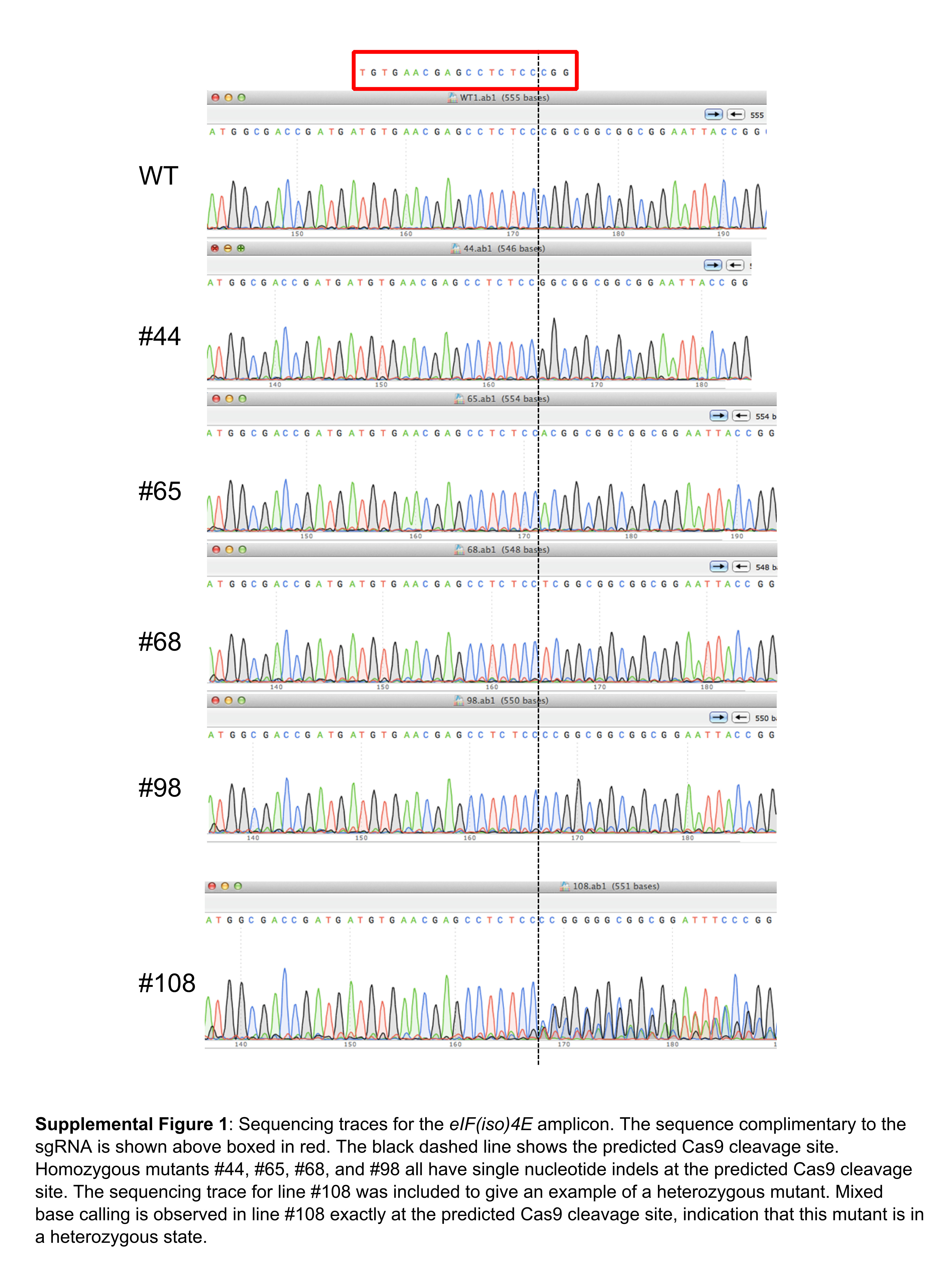

Supplement: Supplementary file 1 — Fig. S1 Sequencing traces for the eIF(iso)4E amplicon. WT, wild‐type. [file MPP-17-1276-s001.jpg]

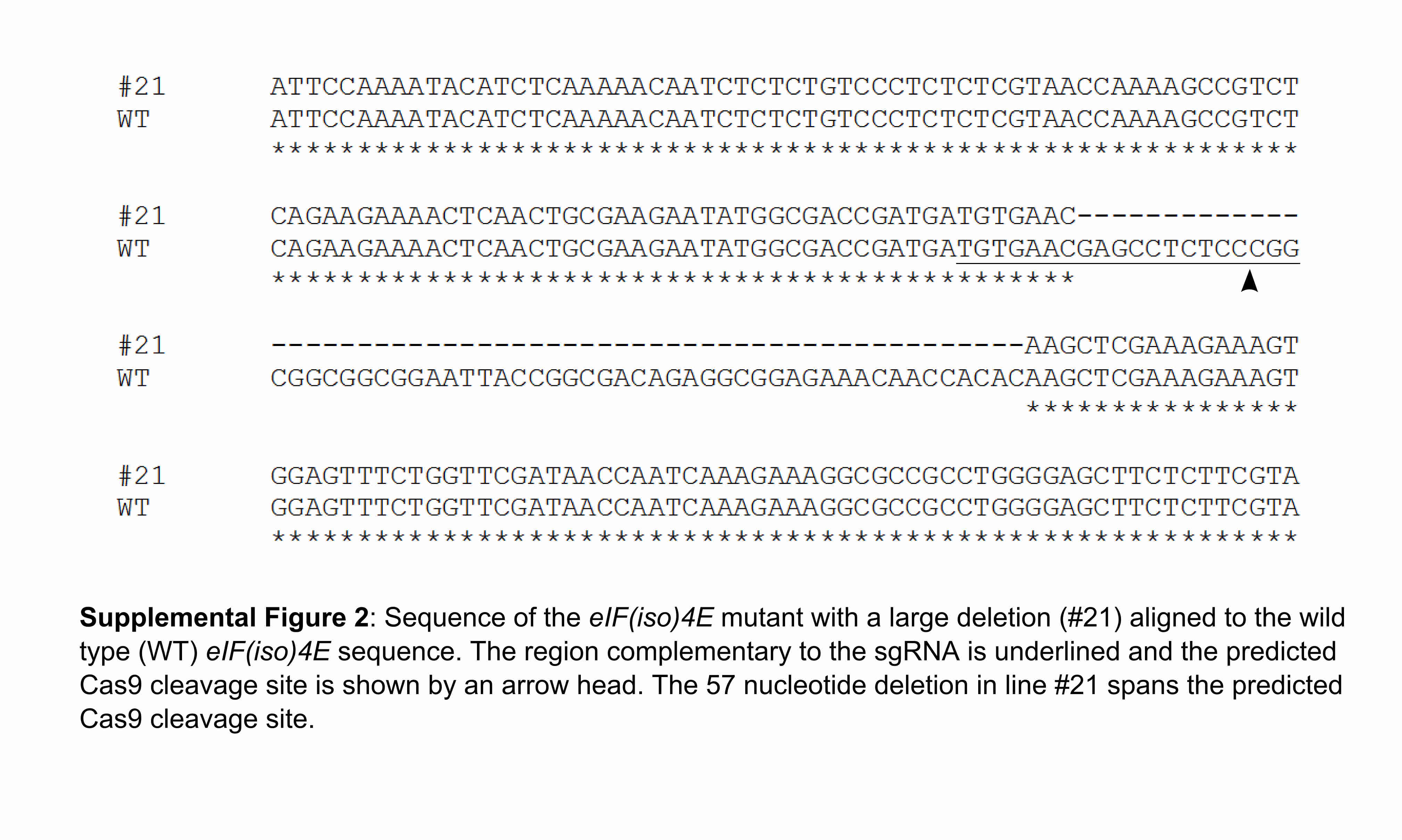

Supplement: Supplementary file 2 — Fig. S2 Sequence of the eIF(iso)4E mutant with a large deletion (#21) aligned to the wild‐type (WT) eIF(iso)4E sequence. [file MPP-17-1276-s002.jpg]

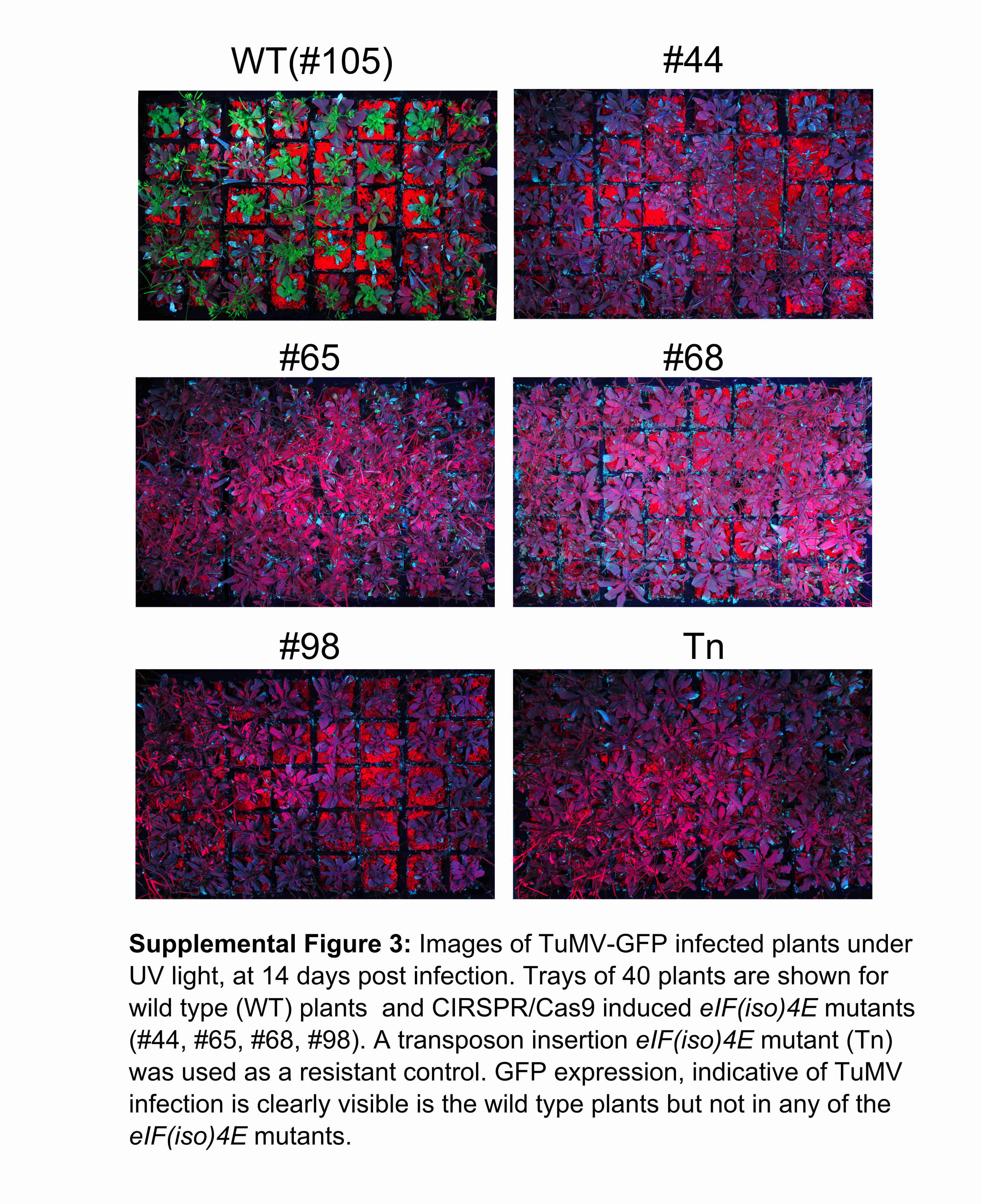

Supplement: Supplementary file 3 — Fig. S3 Images of TuMV‐GFP (green fluorescent protein‐expressing Turnip mosaic virus clone)‐infected plants under UV light at 14 days post‐infection. WT, wild‐type. [file MPP-17-1276-s003.jpg]

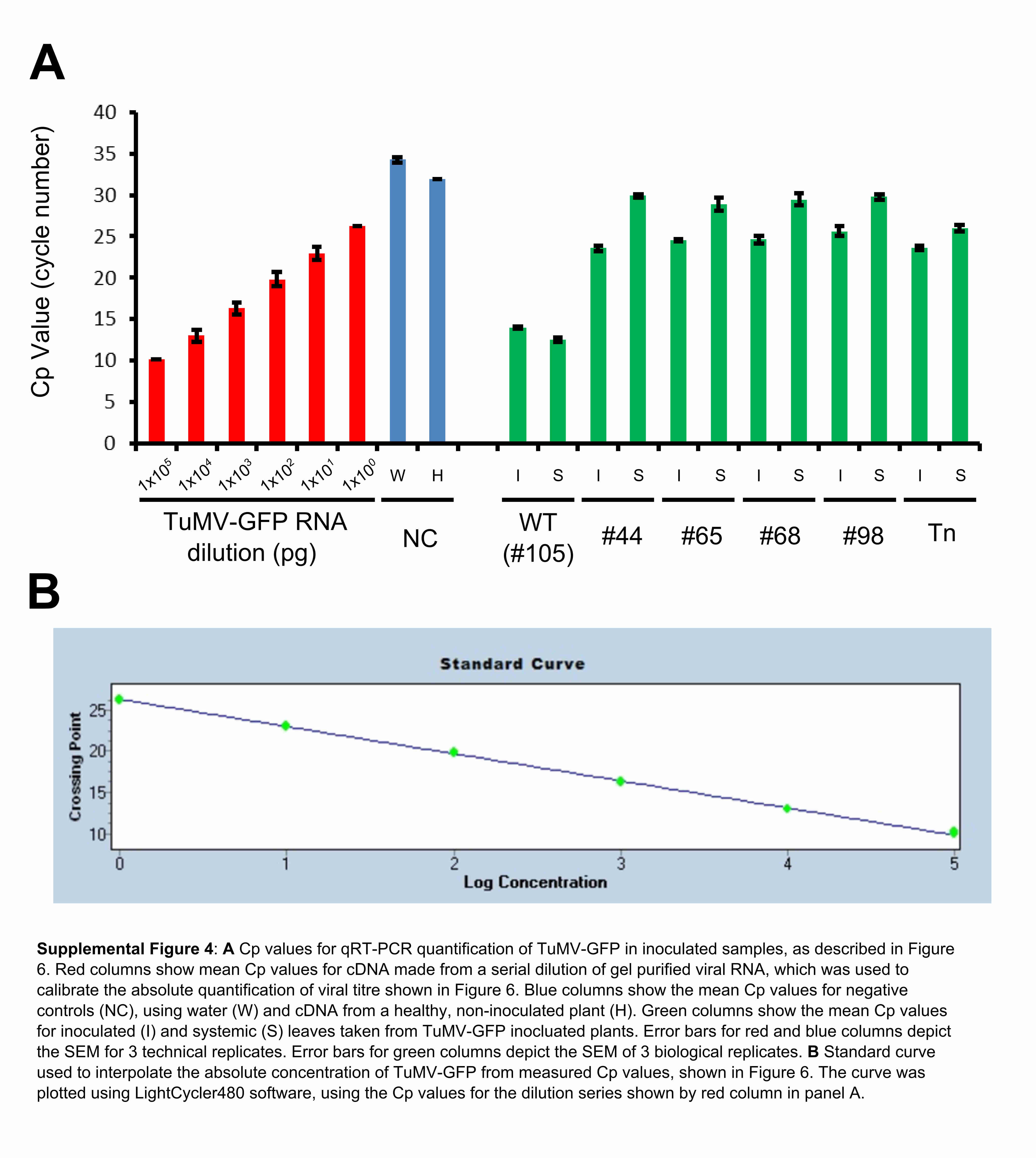

Supplement: Supplementary file 4 — Fig. S4 (A) Cp values for quantitative reverse transcription‐polymerase chain reaction (qRT‐PCR) quantification of TuMV‐GFP (green fluorescent protein‐expressing Turnip mosaic virus clone) in inoculated samples, as described in Fig. 6. (B) Standard curve used to interpolate the absolute concentration of TuMV‐GFP from the measured Cp values, shown in Fig. 6. NC, negative control; WT, wild‐type. [file MPP-17-1276-s004.jpg]
