# Supplementary material for: Engineering of CRISPR/Cas9‐mediated potyvirus resistance in transgene‐free Arabidopsis plants
Source: Mol Plant Pathol. 2016 Jun 27;17(8):1276–88. doi: 10.1111/mpp.12417 (PMC5026172; doi:10.1111/mpp.12417)
Supplement: Supplementary file 5 — Table S1 List of oligonucleotides used in this study, together with the specific polymerase chain reaction (PCR) annealing temperatures used and the expected amplicon sizes. [file MPP-17-1276-s005.docx]

| Oligo Name | Oligo Sequence (5’-3’) | T_a_ (^o^C) | Amplicon size (bp) | Oligo use |
| --- | --- | --- | --- | --- |
| TuMV-CP_Fw | ATGTATGGTCGGTCTTGGTTAC | 53 | 537 | PCR |
| TuMV-CP_Rv | AGAGAAGGCAGAAAAGGAACGA | 53 | 537 | PCR |
| TuMV-CP_qPCR_Fw | GTGGCTCTAAACCTCGATCAT | 60 | 95 | qRT-PCR |
| TuMV-CP_qPCR_Rv | AACCATGTGTCAAACTGCTTTC | 60 | 95 | qRT-PCR |
| eIF(iso)4E _Fw | AAGAGTTAAATGCTCTGATGGAC | 57 | 574 | PCR |
| eIF(iso)4E _Rv | ACAAGTGAATTTCAGCATTCG | 57 | 574 | PCR |
| EF1a_Fw | CTTCTTGAGGCTCTTGACCAG | 59 | 418 | PCR |
| EF1a_Rv | TGAGAGGTGTGGCAATCGAG | 59 | 418 | PCR |
| pCas9_Fw | ATGGATAAGAAGTACTCTATCGGAC | 59 | 664 | PCR |
| pCas9_Rv | GCCTTCTTGACTTAGAGAGCCTAG | 59 | 664 | PCR |
| Iso_Fw | ATTGTGTGAACGAGCCTCTCCCGG | - | - | Cloning |
| Iso_Rv | AAACCCGGGAGAGGCTCGTTCACA | - | - | Cloning |

**Supplemental Table 1**: List of Oligonucleotides used in this study along with the specific PCR annealing temperatures used and expected amplicon sizes.
