# Supplementary material for: Engineering of CRISPR/Cas9‐mediated potyvirus resistance in transgene‐free Arabidopsis plants
Source: Mol Plant Pathol. 2016 Jun 27;17(8):1276–88. doi: 10.1111/mpp.12417 (PMC5026172; doi:10.1111/mpp.12417)
Supplement: Supplementary file 6 — Table S2 Dry weight for wild‐type plants (WT, #105) and homozygous eIF(iso)4E mutant plants (#44, #65, #68, #98). Each dry weight value is the weight of two pooled plants. [file MPP-17-1276-s006.docx]

| Genotype | Dry weight (mg) |
| --- | --- |
| WT | 392 |
| WT | 696 |
| WT | 389 |
| WT | 566 |
| WT | 861 |
| WT | 681 |
| WT | 640 |
| WT | 806 |
| WT | 508 |
| WT | 336 |
| WT | 222 |
| WT | 524 |
| WT | 581 |
| WT | 476 |
| WT | 443 |
| #44 | 687 |
| #44 | 548 |
| #44 | 860 |
| #44 | 850 |
| #44 | 526 |
| #44 | 569 |
| #44 | 659 |
| #44 | 672 |
| #44 | 491 |
| #44 | 445 |
| #44 | 588 |
| #44 | 773 |
| #44 | 614 |
| #44 | 510 |
| #44 | 721 |
| #65 | 548 |
| #65 | 508 |
| #65 | 327 |
| #65 | 605 |
| #65 | 603 |
| #65 | 322 |
| #65 | 519 |
| #65 | 419 |
| #65 | 338 |
| #65 | 793 |
| #65 | 419 |
| #65 | 342 |
| #65 | 372 |
| #65 | 1074 |
| #65 | 605 |
| #68 | 676 |
| #68 | 765 |
| #68 | 940 |
| #68 | 899 |
| #68 | 412 |
| #68 | 744 |
| #68 | 870 |
| #68 | 869 |
| #68 | 345 |
| #68 | 748 |
| #68 | 386 |
| #68 | 358 |
| #68 | 637 |
| #68 | 533 |
| #68 | 434 |
| #98 | 654 |
| #98 | 603 |
| #98 | 569 |
| #98 | 630 |
| #98 | 319 |
| #98 | 567 |
| #98 | 450 |
| #98 | 656 |
| #98 | 493 |
| #98 | 940 |
| #98 | 814 |
| #98 | 349 |
| #98 | 573 |
| #98 | 537 |
| #98 | 759 |

**Supplemental Table 2**: Dry weight for wild type (WT, #105) plants and homozygous *eIF(iso)4E* mutant plants (#44, #65, #68, #98). Each dry weight value is the weight of 2 pooled plants.
