# Supplementary material for: Engineering of CRISPR/Cas9‐mediated potyvirus resistance in transgene‐free Arabidopsis plants
Source: Mol Plant Pathol. 2016 Jun 27;17(8):1276–88. doi: 10.1111/mpp.12417 (PMC5026172; doi:10.1111/mpp.12417)
Supplement: Supplementary file 7 — Table S3 Flowering times for wild‐type plants (WT, #105) and homozygous eIF(iso)4E mutant plants (#44, #65, #68, #98). [file MPP-17-1276-s007.docx]

| Genotype | Flowering time (days) |
| --- | --- |
| WT | 20 |
| WT | 21 |
| WT | 22 |
| WT | 22 |
| WT | 23 |
| WT | 23 |
| WT | 24 |
| WT | 24 |
| WT | 24 |
| WT | 24 |
| WT | 24 |
| WT | 25 |
| WT | 25 |
| WT | 25 |
| WT | 25 |
| WT | 26 |
| WT | 26 |
| WT | 26 |
| WT | 27 |
| WT | 27 |
| WT | 28 |
| WT | 28 |
| WT | 28 |
| WT | 28 |
| WT | 28 |
| WT | 28 |
| WT | 28 |
| WT | 29 |
| #44 | 20 |
| #44 | 21 |
| #44 | 23 |
| #44 | 25 |
| #44 | 25 |
| #44 | 25 |
| #44 | 25 |
| #44 | 25 |
| #44 | 25 |
| #44 | 25 |
| #44 | 25 |
| #44 | 26 |
| #44 | 26 |
| #44 | 26 |
| #44 | 26 |
| #44 | 26 |
| #44 | 27 |
| #44 | 27 |
| #44 | 27 |
| #44 | 27 |
| #44 | 28 |
| #44 | 28 |
| #44 | 28 |
| #44 | 28 |
| #44 | 28 |
| #44 | 28 |
| #44 | 28 |
| #44 | 28 |
| #44 | 28 |
| #44 | 29 |
| #44 | 29 |
| #65 | 20 |
| #65 | 22 |
| #65 | 22 |
| #65 | 22 |
| #65 | 22 |
| #65 | 23 |
| #65 | 23 |
| #65 | 23 |
| #65 | 23 |
| #65 | 23 |
| #65 | 24 |
| #65 | 24 |
| #65 | 24 |
| #65 | 24 |
| #65 | 25 |
| #65 | 25 |
| #65 | 25 |
| #65 | 26 |
| #65 | 26 |
| #65 | 27 |
| #65 | 27 |
| #65 | 27 |
| #65 | 27 |
| #65 | 28 |
| #65 | 28 |
| #65 | 28 |
| #65 | 28 |
| #65 | 28 |
| #65 | 28 |
| #65 | 29 |
| #68 | 21 |
| #68 | 23 |
| #68 | 23 |
| #68 | 24 |
| #68 | 24 |
| #68 | 25 |
| #68 | 25 |
| #68 | 25 |
| #68 | 25 |
| #68 | 25 |
| #68 | 25 |
| #68 | 26 |
| #68 | 26 |
| #68 | 27 |
| #68 | 27 |
| #68 | 28 |
| #68 | 28 |
| #68 | 29 |
| #68 | 30 |
| #68 | 30 |
| #68 | 30 |
| #68 | 30 |
| #98 | 21 |
| #98 | 22 |
| #98 | 23 |
| #98 | 24 |
| #98 | 25 |
| #98 | 27 |
| #98 | 27 |
| #98 | 27 |
| #98 | 28 |
| #98 | 29 |
| #98 | 30 |
| #98 | 31 |
| #98 | 31 |

**Supplemental Table 3**: Flowering times for wild type (WT, #105) plants and homozygous *eIF(iso)4E* mutant plants (#44, #65, #68, #98).
